# Highly variable pharmacokinetics of dexmedetomidine during intensive care: a case report

**DOI:** 10.1186/1752-1947-4-73

**Published:** 2010-02-25

**Authors:** Timo Iirola, Ruut Laitio, Erkki Kentala, Riku Aantaa, Juha-Pekka Kurvinen, Mika Scheinin, Klaus T Olkkola

**Affiliations:** 1 Department of Anaesthesiology, Intensive Care, Emergency Care and Pain Medicine, University of Turku and Turku University Hospital, FIN-20521, Turku, Finland; 2 Department of Pharmacology, Drug Development and Therapeutics, University of Turku and Turku University Hospital, FIN-20521, Turku, Finland

## Abstract

**Introduction:**

Dexmedetomidine is a selective and potent alpha2-adrenoceptor agonist licensed for use in the sedation of patients initially ventilated in intensive care units at a maximum dose rate of 0.7 μg/kg/h administered for up to 24 hours. Higher dose rates and longer infusion periods are sometimes required to achieve sufficient sedation. There are some previous reports on the use of long-term moderate to high-dose infusions of dexmedetomidine in patients in intensive care units, but none of these accounts have cited dexmedetomidine plasma concentrations.

**Case presentation:**

We describe the case of a 42-year-old Caucasian woman with severe hemorrhagic pancreatitis following laparoscopic cholecystectomy who received dexmedetomidine for 24 consecutive days at a maximum dose rate of 1.9 μg/kg/h. Samples for the measurement of dexmedetomidine concentrations in her plasma were drawn at intervals of eight hours. On average, the observed plasma concentrations were well in accordance with previous knowledge on the pharmacokinetics of dexmedetomidine. There was, however, marked variability in the concentration of dexmedetomidine in her plasma despite a stable infusion rate.

**Conclusion:**

The pharmacokinetics of dexmedetomidine appears to be highly variable during intensive care.

## Introduction

Dexmedetomidine is a selective and potent alpha2-adrenoceptor agonist licensed for the sedation of patients initially ventilated in intensive care units (ICU) at a maximum dose rate of 0.7 μg/kg/h administered for up to 24 hours. Higher dose rates and longer infusion periods are sometimes required to achieve sufficient sedation. We describe the case of a 42-year-old Caucasian woman with severe hemorrhagic pancreatitis following laparoscopic cholecystectomy, who received dexmedetomidine for 24 consecutive days at a maximum dose rate of 1.9 μg/kg/h.

## Case presentation

A 42-year-old Caucasian Finnish woman was scheduled for laparoscopic cholecystectomy due to typical symptoms and radiological findings of gallstones. She was obese (89 kg, BMI = 33), even though she had managed to lose weight by 20 kg six months prior to presentation. She was using sibutramine and oral contraceptives as regular medication.

Surgery was uneventful, but on the second postoperative day, the general state of our patient started to deteriorate, resulting in anuria and difficulty of breathing, admission into the intensive care unit (ICU), endotracheal intubation, and mechanical ventilation. Endoscopic retrograde cholangiopancreatography (ERCP) was performed upon ICU admission because of suspected biliary tract leakage. However, no signs of leakage were observed. Computed tomography (CT) examination revealed fluid around her liver, while her pancreas could not be visualized. Her plasma amylase concentration was elevated, thus confirming the diagnosis of pancreatitis.

Due to decreased renal function, she was commenced on continuous hemodiafiltration therapy on the third day and continued until the 10th postoperative day. Propofol infusion for sedation, supplemented with intravenous oxycodone boluses, was started as part of our hospital's standard therapy in order to facilitate mechanical ventilation and other treatment procedures. Propofol sedation was continued until the 36th postoperative day. Upon the decision of weaning, her attending physician decided to add dexmedetomidine infusion into the sedation regimen 17 days after her surgery.

Weaning was not successful, and tracheostomy was performed on the 18th postoperative day. On the 19th postoperative day, esophagogastroduodenoscopy and explorative laparotomy were performed because the general condition of our patient again started to deteriorate. Hemorrhagic pancreatitis with severe inflammation of her abdominal cavity was discovered. This deterioration prevented further weaning. Our aim was to stop propofol infusion after starting her on dexmedetomidine, but in order to achieve the desired level of sedation (light to moderate, Sedation-Agitation Scale (SAS) levels 3 to 4 [1]), propofol infusion had to be continued despite the already high dose of dexmedetomidine she was receiving.

Our patient later required yet another laparotomy because of elevated abdominal pressure. This surgery did not reveal any additional findings, but this time her abdominal wall had to be left open, and sedation had to be continued. Vacuum-assisted closure therapy was established on the 22nd and continued until the 34th postoperative day. Weaning was started again on the 36th postoperative day. Clonidine infusion was started on the 40th postoperative day, while the dexmedetomidine infusion was discontinued on the following day, on the 24th day of its administration.

Our patient's abdominal wall was finally closed on the 47th day, and the clonidine infusion was stopped on the 51st postoperative day. The tracheostomy cannula was removed on the 54th day and she was admitted to a surgical ward on the 55th postoperative day for further recovery.

During dexmedetomidine sedation, her plasma albumin level was low (8.4 g/L to 11.6 g/L) and her creatinine level was slightly elevated (23 μmol/L to 126 μmol/L). Meanwhile, her bilirubin level and international normalized ratio (INR) were both normal at 5 μmol/L to 16 μmol/L and 1.1 to 1.4, respectively.

At 14 months later, her recovery is still incomplete. Already in the ICU she complained that her vision is impaired. According to the consulting ophthalmologist, this symptom is likely due to ischemic optic neuropathy. At present she can only see movement and light with her right eye. The vision of her left eye is also severely impaired, but she is able to read using special equipment for the visually impaired. Additionally, her everyday life is harmed by numbness and weakness of her extremities, which is caused by critical illness polyneuropathy. Despite these impairments, her aim is to return to work.

Samples for the measurement of dexmedetomidine concentrations in her plasma were drawn at 8-hour intervals as directed by the plan of the pharmacokinetic study she was recruited in. The concentrations were determined using reversed-phase high-performance liquid chromatography with tandem mass spectrometric detection (PE Sciex API4000 instrument; PE Sciex, Foster City, California, US) as described previously [2].

**Figure 1:**
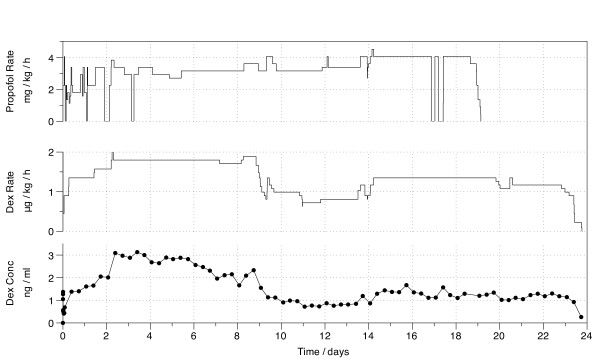
**Dexmedetomidine infusion rate and plasma concentrations**. Propofol infusion rate, dexmedetomidine infusion rate and plasma dexmedetomidine concentration during the 24-day high-dose infusion in a critically ill intensive care patient (Dex, dexmedetomidine; Conc, concentration).

The rates of the dexmedetomidine and propofol infusions, as well her plasma concentration results of dexmedetomidine, are presented in Figure 1. In calculating the dexmedetomidine dose, we used her preoperative weight of 89 kg.

Because the dose rate of dexmedetomidine remained constant for relatively long periods of time during three separate intervals, we calculated the plasma clearance of dexmedetomidine during these intervals by dividing the infusion rate by the plasma concentration. The calculated clearance was 55 L/h, 92 L/h and 87 L/h during the 2nd to 6th, 14th to 20th and 21st to 23rd day of the dexmedetomidine infusion, respectively. A list of drugs administered during her ICU stay is presented in Table 1.

**Table 1:** Drugs administered during our patient's stay at the intensive care unit.

	Start (day)	Stop (day)	Dosage
**Regularly administered drugs**			
Calcium glubionate	3	21	90 mg × 2 - 6 iv
Enoxaparin	3	9	20 mg to 40 mg when needed for CVVHDF
	10	54	40 mg × 1 sc
Sodium polystyrene sulfonate	3	3	30 g × 1 pr
Pantoprazol	3	54	40 mg × 1 iv
Imipenem	3	39	250 mg to 1000 mg × 3 iv
Ondansetron	3	3	4 mg × 2 iv
Lactulose	6	9	20 g × 3 po
Vancomycin	8	54	1000 mg × 2 iv (based on the serum concentration)
Metronidazole	11	19	400 mg × 3 po
	19	26	500 mg × 3 iv
Fluconazole	12	51	400 mg × 1 iv
Hydrocortisone	13	15	100 mg × 3 iv
Ciprofloxacin	18	39	400 mg × 2 iv
Tigecycline	39	54	50 mg × 2 iv
			
**Infusions**			
Short-acting insulin	3	54	0.5 - 20 IU/h
Furosemide	3	3	1000 mg/day
	6	36	70 - 1000 mg/day
Norepinephrine	3	31	Maximum dose 0.19 μg/kg/min
Propofol	3	36	See Figure 1.
Dexmedetomidine	17	41	See Figure 1.
Metoprolol	38	40	1 - 2 mg/h
Clonidine	40	51	Maximum dose 2.2 μg/kg/h
			
**Drugs administered when needed**			
Propofol	3	37	20 - 50 mg iv
Diazepam	3	3	2.5 mg iv
Lorazepam	26	41	1 - 4 mg iv
Haloperidol	36	55	2.5 - 5 mg iv
Fentanyl	3	31	Occasional doses of 50 - 200 μg iv
Oxycodone	3	53	5 - 10 mg iv
Acetaminophen	11	21	Occasional doses of 1000 mg po/iv
Furosemide	3	52	5 - 10 mg iv
Ephedrin	3	3	5 mg iv
Rocuronium	3	32	Occasional doses of 10 - 50 mg iv
Indapamide	15	15	2.5 mg × 1
Metoprolol	26	42	Occasional doses of 2.5 - 5 mg iv

Days refer to postoperative days. IV, intravenous; po, per os; pr, per rectum; IU, international units; CVVHDF, continuous veno-venous hemodiafiltration.

## Discussion

There are some previous reports on the use of long-term moderate to high dose infusions of dexmedetomidine in ICU patients [3], but none of these accounts have reported dexmedetomidine plasma concentrations. In our patient, the infusion rates were higher than recommended, and her dexmedetomidine plasma levels were measured over a 3-week infusion period. On the average, our patient's observed plasma concentrations were well in accordance with previous knowledge on the pharmacokinetics of dexmedetomidine in humans [4]. However, the concentration of our patient's dexmedetomidine greatly varied even during unchanged infusion.

The plasma concentration of dexmedetomidine decreased by one-third (2.9 ng/ml to 1.7 ng/ml) on days 6 to 8 despite a constant rate of infusion. The concentration of any drug at a steady state is dependent only on its plasma clearance and the rate of infusion. Accordingly, the calculated clearance of dexmedetomidine was increased by 60%. The reason for the increased clearance can only be speculated.

Dexmedetomidine is almost completely eliminated by metabolism in the liver. It is mainly N-glucuronidated by glucuronyl transferases and hydroxylated by several cytochrome P450 enzymes [5], but none of the drugs which were administered at the time of the apparent change in dexmedetomidine clearance are known to induce the activities of glucuronyl transferases or cytochrome P450 enzymes. It is thus logical to assume that changes in hemodynamic variables could have affected the pharmacokinetics of dexmedetomidine.

Although there is no direct information on the extraction ratio of dexmedetomidine in humans, the reported values of dexmedetomidine clearance (40 L/h to 70 L/h in adults) [4] suggest that the extraction ratio of dexmedetomidine is rather high, and its clearance may thus be dependent on liver perfusion. This hypothesis is supported by data from Ebert *et al*. [6] and Snapir *et al*. [2] who observed that high-dose target controlled infusions of dexmedetomidine produced higher plasma concentrations than expected, probably due to decreased cardiac output caused by dexmedetomidine itself. Unfortunately, we have no data from our patient on cardiac output or intestinal perfusion at the time of major changes in her dexmedetomidine clearance. Nevertheless, the increase in apparent dexmedetomidine clearance coincided with the general improvement of the condition of our patient. For instance, the dose rate of norepinephrine required to maintain her hemodynamic function was significantly reduced on the 5th day of the dexmedetomidine infusion.

Although the dose rate of dexmedetomidine was high, its sedative effect had to be enhanced with propofol. It is quite common that several different types of drugs acting via different mechanisms are combined during long-term ICU treatment. Our patient was commenced on clonidine because clonidine was routinely used to facilitate the termination of long sedation or opioid infusions at the time of the study. However, in the case of dexmedetomidine, the change to another alpha2-adrenoceptor agonist was probably unnecessary.

Our patient developed optic neuropathy probably because of cerebral ischemia secondary to hypotension, hypoxia or embolism. Although a toxic mechanism cannot be excluded, we have no reason to believe that this complication was due to dexmedetomidine. There is a plethora of underlying conditions for ischemia during critical illness and there are no previous reports of toxic neuropathy following dexmedetomidine infusion.

## Conclusion

During our patient's 24-day high-dose dexmedetomidine infusion, her observed plasma concentrations were on the average well in accordance with previous knowledge on the pharmacokinetics of dexmedetomidine in humans. There was, however, a marked variability in the concentration of dexmedetomidine in her plasma despite a stable infusion rate. We conclude that the pharmacokinetics of dexmedetomidine appears to be highly variable during intensive care. However, the pharmacokinetics of dexmedetomidine appears to be linear even at high-dose and long-lasting administration. We observed no unexpected accumulation of dexmedetomidine during the infusion.

## Consent

Written informed consent was obtained from the patient for publication of this case report and accompanying images. A copy of the written consent is available for review by the Editor-in-Chief of this journal.

## Competing interests

TI, RL, EK, RA and KTO have ongoing contract research relationships with Orion Corporation (Espoo, Finland), the original developer of dexmedetomidine.

TI has received speaker fees from Orion Corporation.

RA has been a paid consultant for Orion Corporation and Abbott Laboratories (Abbott Park, Illinois, US), the original co-developers of dexmedetomidine, as well as for Hospira (Lake Forest, Illinois, US). Hospira has a license agreement with Orion Corporation concerning dexmedetomidine (Precedex^®^).

JPK has been engaged in contract research for Orion Corporation and Hospira.

The laboratory of MS has contract research relationships with Orion Corporation and Hospira. Hospira has a license agreement with Orion Corporation concerning dexmedetomidine (Precedex^®^). MS has also received speaker fees and consulting fees from Orion Corporation.

## Authors' contributions

TI, RL and EK were involved in patient care and collected her blood samples. JPK and MS analyzed the samples. TI, RL, EK, RA, JPK, MS and KTO were involved in the interpretation of data and review of literature. They also drafted and revised the manuscript. All authors read and approved the final manuscript.
